# Use of Virtual Reality in Children with Dyslexia

**DOI:** 10.3390/children9111621

**Published:** 2022-10-25

**Authors:** Giuseppa Maresca, Simona Leonardi, Maria Cristina De Cola, Silvia Giliberto, Marcella Di Cara, Francesco Corallo, Angelo Quartarone, Alessandra Pidalà

**Affiliations:** IRCCS Centro Neurolesi Bonino Pulejo, 98123 Messina, Italy

**Keywords:** dyslexia, virtual reality, learning disabilities

## Abstract

In recent years, the study of dyslexia has seen rapid progress in definition and classification, neuropsychological correlates, neurobiological factors, and intervention. However, there are few studies on how virtual reality can affect improving cognitive domains and cross-cutting pedagogical skills. We, therefore, tested intervention through the use of a virtual reality rehabilitation system (VRRS) in children with dyslexia. Twenty-eight patients diagnosed with dyslexia were enrolled in this study. One-half underwent conventional neuropsychological treatment, and the other half performed VR neurorehabilitation training using the VRRS. All patients were evaluated by neuropsychological assessment at baseline (T0) and at the end of the protocol (T1). The assessment included the administration of the Wechsler Intelligence Scale for Children-IV and the Italian Battery for the Assessment of Dyslexia and Dysorthography. Our results showed a significant difference in word-reading test scores as well as in homophonic writing. In addition, treatment type was found to affect some domains of the WISC. We believe that the VRRS led to improved outcomes through the use of VR, which encourages active exploration, improves engagement, and provides motivation and enjoyment, allowing longer training sessions and improving treatment adherence.

## 1. Introduction

Over the past 25 years, the scientific understanding of dyslexia and other learning difficulties has seen rapid advances in areas involving their definition and classification, neuropsychological correlates, neurobiological factors, and intervention [1].

Dyslexia is a reading disorder in children and adults, characterized by partial deficits in the reading and spelling of a single word [2,3]. Prevalence estimates range from 6% to 17% of the school-age population, depending on the criteria for the severity of reading difficulties [4]. There is a higher incidence in males, with a ratio of about 1.5:1, but this is lower than the historical estimates of about 3.4:1 [5]. The basis of dyslexia is neuro-biological, with significant evidence of heredity; dyslexia can be remedied in many children through early rehabilitation [3,4]. It is now well established that dyslexia is a neurological disorder of genetic origin, currently under investigation. Beyond this consensus, the biological and cognitive causes behind reading delays are still debated [6,7,8,9].

Although there is now a strong consensus among researchers that the central difficulty in dyslexia reflects a deficit within the language system (the phonological theory) [10,11,12,13], other theoretical models remain convincing such as the theory of auditory temporal processing deficits [14], the cerebellar theory [15], and, more recently, the visual attention deficit theory [16,17,18,19], and the magnocellular visual impairment theory of dyslexia [20,21,22]. The last of these postulates that the magnocellular pathway is selectively disrupted in some dyslexic individuals and that this leads to deficiencies in visual processing. The visual theory does not rule out a phonological deficit but emphasizes a further visual contribution to reading problems, at least in some dyslexic individuals [23].

The different patterns of performance observed have led various researchers to consider developmental dyslexia a heterogeneous disorder resulting from independent cognitive deficits, with a majority subtype affected by phonological deficits and a minority subtype characterized by visual deficits [18,24]. Many authors in the optometric literature, contrary to the ophthalmological literature, defend the view that children with reading disorders have an increased incidence of vision abnormalities and proclaim the usefulness of vision therapy for reading and learning difficulties [25,26], although it has not been demonstrated that there is a significant difference in reading ability between readers with normal and abnormal binocular function [27]. Other studies have also not been able to find an increased incidence of binocular disorders in children with reading difficulties or an association between motility disorders and reading skills [28].

An effective method for rehabilitation is virtual reality (VR), a tool that includes a set of methods that retrain or alleviate the problems caused by attention, visual processing, language, memory, reasoning, resolution of problems, and deficits in executive functions.

One system that has been developed in recent years is the VR rehabilitation system (VRRS), which has cognitive and language-related modules aimed at improving cognitive and language deficits in patients with neurological impairments [29,30]. There are no studies on the effects of the virtual reality rehabilitation system on children with dyslexia. Virtual reality can be very useful when applied to pupils with dyslexia problems as it reduces performance anxiety, facilitates the visualization of texts, and fosters greater motivation: immersing oneself in a parallel reality increases engagement and makes the perceived complicated activities more attractive and less boring. In addition, virtual reality has the power to expand the experience, making one experience situations that would not be possible in normal reality.

This feature will also be exploited so that other people, such as teachers and classmates, can experience first-hand the difficulties that pupils with dyslexia face in reading and consequently learn about their frustrations and emotional state. We expect this information to be used by academics to expand their teaching methodology. In fact, one of the objectives is to try, in small steps, to use all the information gathered to encourage the adoption of a new teaching method that is suitable for all students [31].

We believe that this approach can have positive effects on the student’s motivation, anxiety management, and sense of efficacy. Inclusion also starts from here: from stimulating, reflecting, and studying policymakers, with an impact not only on the individual but also on the societal level. However, the literature needs to grow in order to give more scientific validity to these methods [32].

The aim of our study is to verify the intervention through the use of a VRRS in children with dyslexia, hypothesizing an additional improvement at the cognitive level.

## 2. Materials and Methods

### 2.1. Study Population

Twenty-eight patients (15 females and 13 males) with a diagnosis of dyslexia (mean ± SD age: 10.3 ± 2.0 years), admitted to the IRCCS Centro Neurolesi “Bonino Pulejo” of Messina, were enrolled in this study and randomized into either the control group (CG: *n* = 14) or the experimental group (EG: *n* = 14). A more detailed description of these groups is in Table 1.

The inclusion criteria were: (1) a diagnosis of dyslexia according to the Diagnostic Statistical Manual, Fifth Edition (DSM-5). The DSM-5 diagnostic criteria for SLDs requires that the child fulfill the following four criteria: (a) the child had at least six symptoms of learning difficulties during the period of at least 6 months despite the provision of extra help or targeted instruction, (b) the child with SLDs usually has difficulties in literacy and mathematical skills such as reading a single word, reading comprehension, writing and spelling, arithmetic calculation, and mathematical reasoning; (c) the deficits in keystone academic skills have led to poor academic achievement and the child tends to lag far behind in age and intellectual ability from their peers; and (d) the lag in academic achievement is not due to intellectual disabilities, other mental or neurological disorders, visual or auditory problems, or poor or inappropriate academic instruction (American Psychiatric Association 2013; Diagnostic and Statistical Manual of Mental Disorders, 5th ed, Washington). (2) The absence of severe medical and psychiatric illness. The local ethics committee approved the study, and all subjects were informed and gave their written consent to study participation and publication. 

### 2.2. Design of the Study

It was a prospective, rater-blinded longitudinal study lasting around 6 months. The CG underwent conventional neuropsychological treatment (CNT), and the EG performed VR neurorehabilitation training (VRNT) using the virtual reality rehabilitation system (VRRS). All patients were assessed by means of neuropsychological evaluation at the beginning and at the end of each rehabilitative program, during which they underwent a total of 72 training sessions of 1 h duration, three times a week. All patients with a diagnosis of dyslexia were assessed by means of neuropsychological evaluation at baseline (T0) and at the end of the protocol (T1). 

### 2.3. Neuropsychological Assessment

Neuropsychological evaluation was performed by a skilled neuropsychologist by means of the administration of the Wechsler Intelligence Scale for Children-IV (WISC-IV) [33] and the Italian Battery for Evaluation of Dyslexia and Dysorthography (DDE) [34]. WISC-IV is a clinical tool that allows us to evaluate the cognitive abilities of children and young people aged between 6 and 16 years and 11 months. WISC-IV evaluates four cognitive areas using specific cognitive indices: the Verbal Comprehension Index (VCI), the Visual–Perceptual Reasoning Index (PRI), the Working Memory Index (WML), and the Processing Speed Index (PSI). In addition, WISC-IV provides three composite indices: the Global Intellectual Quotient (IQ), the General Ability Index (IAG), and the Cognitive Competence Index (ICC). WISC-IV is useful for the cognitive assessment of children with specific learning disorders (SLDs) in order to support the diagnostic hypothesis through a standardized test. In Italy, in fact, an IQ of at least 85 and a significant discrepancy between the IQ and the academic performance affected by the disorder are required to diagnose an SLD [35]. The structure of WISC-IV pays greater attention to working memory and processing speed and makes this tool useful for diagnosing SLDs. Indeed, much research has shown that SLDs are associated with impaired performance in these two cognitive functions (e.g., [36,37]). The DDE battery allows us to evaluate the level of competence acquired in reading and writing and to monitor its progress to compare diagnosis and treatment results. The DDE includes 8 tests, 5 for the analysis of the reading process (naming of graphemes, reading of words and non-words, understanding of sentences with homophones, correction of homophones) and 3 for the analysis of the writing process (dictation of words and non-words, dictation of sentences with homophonic words). It is useful for deepening reading and writing difficulties during a diagnosis of SLDs, checking the evolution of reading and writing systems, and comparing diagnosis and treatment results by promoting communication between operators and rehabilitation centers. The DDE is included in the basic diagnostic protocol for the assessment of learning disorders of reading, writing, and calculation, approved by the Italian Dyslexia Association. 

### 2.4. Virtual Reality Neurorehabilitation Treatment (VRNT) with Khymeia VRRS

VRNT was conducted by means of the virtual reality rehabilitation system (VRRS, Khymeia, Padua, Italy), a tool used in clinical practice to rehabilitate and tele-rehabilitate a wide spectrum of pathologies. The VRRS allows the multisensory and interactive simulation of scenarios that concern real life with the aid of a computer. The recreated situations are generally three-dimensional and reproduce real objects and events, improving the cognitive abilities of patients [38]. The VRRS represents a clinical and technological innovation, allowing the therapist to customize the rehabilitation process for each patient by establishing the type, difficulty, and duration of the exercises. The integration of the various rehabilitation modules allows us to adapt the rehabilitation to the real needs of the patient in a simple and rapid way. The VRRS cognitive module used in this study consists of a wide range of rehabilitative activities, with more than fifty exercises already available and many others under development. All activities are organized to stimulate the different cognitive domains: memory, attention, language, spatial–temporal orientation, executive functions, calculation, and practice. The cognitive exercises consist of 2D exercises in which the patient interacts with objects and scenarios through the touch screen or with a particular magnetic sensing sensor paired with a compressible object, such as a mouse, thus emulating the ability of interaction. All the virtual exercises have been planned and organized by the therapist (after consultation with the neuropsychiatrist), with increasing difficulty in relation to the time of execution and the type of activity. The VRRS is designed to allow increased feedback to the central nervous system through intensive, repetitive, and task-oriented exercises that are performed in a virtual environment, hence developing knowledge of the results and the quality of the movements (knowledge of the performance). In fact, this can activate “reinforcement learning” that encourages an increase in information on a movement, hence obtaining an improvement in the quality of the performances [30,39]. Moreover, training in a playful VR environment could be more motivating for patients, and motivation is the basis for a more successful recovery.

### 2.5. Statistical Analysis

The Mann–Whitney U-test and the Fisher exact test were used to compare demographic and clinical variables between the two groups, where appropriate. An analysis of covariance (ANCOVA) was performed to evaluate whether the means of the clinical outcome at follow-up (dependent variable) are equal across levels of the treatment (categorical independent variable) while statistically controlling for the effects of another continuous variable (covariate). Notably, the model had the test score at T1 as the dependent variable, the binary variable ‘Group’ (EG; CG) as the independent variable, and the test score at T0 as the covariate. Both the assumption of homogeneity of regression slopes as well as the homogeneity of the variance assumption were assessed by ANOVA and Levene’s test, respectively. The F-statistic and the adjusted R2 of the ANCOVA model were used as standardized measures of effect sizes. Data were analyzed using R version 4.0.5, considering a *p*-value <0.05 as statistically significant.

## 3. Results

No significant differences in demographic characteristics between the groups were found (Table 1). Similarly, the two groups did not show significant differences in pedagogical tests at baseline. However, at the end of the study, a significant difference in the word-reading test scores (*p* = 0.019) as well as in homophone writing (*p* = 0.034) was found.

**Figure 1:**
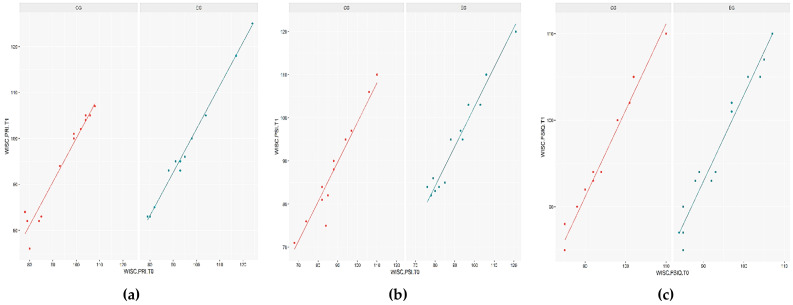
Plot of the predicted values from the covariate models, for each group (EG = Experimental group; CG = Control group): (**a**) covariate = PRI score at T0; outcome variable = PRI score at T1; (**b**) covariate = PSI score at T0; outcome variable = PSI score at T1; (**c**) covariate = FSIQ score at T0; outcome variable = FSIQ score at T1.

Both the assumptions of homogeneity of regression slopes and covariate-treatment independence were tenable in all covariate models. The interaction term was not considered in the ANCOVA models’ fitting because ANOVA has shown that this term does not bring significant information to the covariate models. As visible in Table 2 and Figure 1a–c, after controlling for the effect of the scores at baseline, we found that the type of treatment affected the WISC domains of PRI (*t* = 2.809; *p* < 0.01), PSI (*t* = 3.352; *p* < 0.01), and FSIQ (*t* = 3.071; *p* < 0.01), increasing the scores of these tests in the experimental group significantly. 

## 4. Discussion

Virtual reality is an innovative tool that, due to its multisensory and immersive nature, can fulfill the principles of active learning. Indeed, immersive virtual experiences foster a sense of presence and embodiment, both of which are key factors that can promote learning. The use in education of so-called immersive devices—there are different types and different levels of involvement—is still in its infancy, and there are, of course, pros and cons, for and against. Let us take stock, starting with a fact: immersed in a digital society, as we all are, the student can no longer be considered a passive receiver who acquires knowledge simply by observing or listening to something of interest. However, when it comes to education, we generally continue to imagine children sitting at their desks, intent on reading some textbook or listening to the teacher talking about the French Revolution or Newton’s law or other topics from a wide variety of subjects. The general idea is that of a student receiving information from third parties—be it the teachers, the books, or the documentaries that are watched in class. However, the latest research shows a very different reality [40].

Currently, different types of treatment or intervention programs are available to address the symptoms of dyslexia in children [41]. Of utmost importance is the assessment of the etiology of the disorder in order to plan the intervention appropriately. Indeed, it is useful for any intervention to also take into account possible comorbidities in order to be as comprehensive as possible [42].

Pecini et al. [43], through their studies, found that the use of virtual reality could be a rehabilitation option for children with reading difficulties, improving the cognitive processes underlying reading. This could be useful for the implementation of intensive, specific, and early interventions that, in the traditional approach, involve a number of complications. VRRSs can engage diverse linguistic, visual, and attentional processes and integrate the components into a complex task, such as reading. According to these studies, the use of virtual reality may be a promising approach that can potentially address multiple cognitive and linguistic components underlying normal and impaired reading, as dyslexia has a “multifunctional deficit model,” facilitating the automation of reading processes [44,45].

The aim of our study was to verify the intervention through the use of a VRRS in children with dyslexia, hypothesizing an additional improvement on the cognitive level. 

The results showed an improvement in the test sample compared to the control sample in the specific cognitive domains of the WISC, including the PRI, PSI, and FSIQ. Cross-cutting the cognitive improvement in the specific domains, a significant increase was also found in word-reading test scores as well as in homophonic writing. The development of different cognitive skills is a crucial goal in the rehabilitative treatment of these patients and may represent the first step toward phonological awareness and improved decoding skills. 

Our results suggest that intervention with VRRSs has multiple benefits for patients with SLD issues. This finding is critical as there is no pharmacological therapy for SLDs; there is a need for the use of non-pharmacological interventions that can help improve cognitive performance and not just supplement traditional therapy.

Overall, our findings are in line with several studies that have been conducted on dyslexia [46] or other conditions that present cognitive difficulties, such as stroke survivors, people with Parkinson’s disease, and MS [47,48].

We believe that the VRRS has led to improved outcomes through the use of VR, which encourages active exploration, improves engagement, and provides motivation and enjoyment, allowing longer training sessions and improving treatment adherence.

Thus, we can argue that the use of telerehabilitation using VR for dyslexia is feasible and effective as it allows us to enhance the rehabilitation process, increasing the recovery of language skills in addition to cognitive functions. 

## 5. Conclusions

In conclusion, this study shows that the application of VRRS rehabilitation programs could be one of the solutions to treating children with dyslexia, classifying it as a promising treatment (also for monitoring the results) to maintain and/or enhance language skills, reduce disability, and promote psychological well-being. More studies are needed to clarify the effect of VRRSs on deficits associated with dyslexia; the results of our study are promising, although the small sample size highlights the need for further study.

## Figures and Tables

**Table 1:** Sociodemographic description of the sample.

	*All*	*EG*	*CG*	*p-Value*
Participants	28	14 (50.0)	14 (50.0)	-
Male	13 (46.3)	7 (50.0)	6 (42.9)	0.99
Age (years)	10.3 (2.0)	10.5 (2.1)	10.1 (2.0)	0.61
Education (years)	5.2 (1.9)	5.4 (1.8)	5.1 (2.0)	0.67

Legend: Experimental Group (EG); Control Group (CG). Continuous variables were expressed as mean (standard deviation), whereas categorical variables as frequencies (percentages).

**Table 2:** ANCOVA results for each covariance model on WISC domains.

Clinical Assessment	Group Coefficient				Adjusted R^2^
	Estimate	Std. Error	t Value	*p* Value	
**VCI**	0.355	0.579	0.613	0.545	0.976
**PRI**	1.467	0.522	2.809	**0.009**	0.970
**WMI**	0.240	0.569	0.422	0.676	0.956
**PSI**	2.560	0.764	3.352	**0.002**	0.943
**FSIQ**	1.263	0.411	3.071	**0.005**	0.965

Significant differences between treatment effects are in bold. Legend: VCI = Verbal Communication Index; PRI = Perceptual Reasoning Index; WMI = Working Memory Index; PSI = Processing Speed Index; FSIQ = Full Scale IQ.

## Data Availability

Not applicable here.
